# Survival from laryngeal cancer in England and Wales up to 2001

**DOI:** 10.1038/sj.bjc.6604582

**Published:** 2008-09-23

**Authors:** C M Nutting, M Robinson, M Birchall

**Affiliations:** 1 Department of Radiotherapy, Head and Neck Unit, Royal Marsden Hospital, Fulham Road, London SW3 6JJ, UK; 2 Department of Radiotherapy, Weston Park Hospital, Witham Road, Sheffield, UK; 3 Department of Surgery, Clinical Sciences at South Bristol, Faculty of Medicine and Dentistry, University of Bristol, Bristol, UK

Head and neck cancers arise from the epithelium of the upper airway and digestive tracts. The term incorporates cancers of the mouth, larynx, throat, nose, ears, salivary glands, thyroid or neck, which together make up about 6% of all cancers in the United Kingdom and half a million cases worldwide *per annum*. The UK incidence is 8–12 (depending on region) cases per 100 000 population *per annum*, or about 6500 new cases. They are more common in men than women, and the dominant aetiological associations are with tobacco and alcohol consumption.

Cancer of the larynx is the most common type of head and neck cancer in most western countries. Coleman *et al* (2004) reported 5666 cases in men in the United Kingdom in the 3-year period between 1996 and 1999. These authors also described a 3.3% improvement in survival every 5 years over the period of study between 1986–1999 and 1996–1999. Most UK patients present early, as even a small growth on the vocal cords (73% occur here) causes a discernible change in voice. However, around 30% present at a later stage, either because voice change was ignored or because of lack of recognition of the much vaguer symptoms associated with tumours beyond the vocal cords.

Treatments include surgery, radiation, chemotherapy or combinations of these three. According to the second DAHNO report (Wight, 2008), 63% of early vocal cord cancers still received radiotherapy in the United Kingdom in 2006, although endoscopic resection techniques had significantly gained popularity since the first report (for 2004) when the figure for radiotherapy was 77%. In 2006, 67 of 69 early vocal cord cancer patients reported to have had surgery were treated endoscopically. In the same report, 61% of advanced lesions were treated with a combination of surgery and radiotherapy.

## Changes in practice between 1985 and 2001

Significant advances in the period 1985–2001 included improvements in diagnostic techniques: routine use of the flexible laryngoscope in ENT outpatients, advances in computed tomography and magnetic resonance imaging. Therapeutic advances include the more widespread acceptance of combined modality therapy with surgery and the application of postoperative radiation for advanced laryngeal tumours. As demonstrated by the DAHNO audits (Wight, 2008), the European enthusiasm for endoscopic excision of laryngeal cancer has gradually spread to the United Kingdom over the last 5 years, though it is still largely restricted to early-stage disease. The use of selective neck dissection in combination with laryngectomy may have reduced loco-regional recurrence rates, but the biggest improvements in surgical treatment are in the area of rehabilitation and particularly the use of prosthetic voice valves. The widespread application of CT planning has significantly improved the quality of radiotherapy delivery for head and neck cancer. Supported by some large recent trials, concomitant chemoradiation therapy is increasingly used for stage III and IV disease, and postoperatively in those with poor prognostic features and who are fit enough for this arduous combination. Despite the increasingly widespread use of chemoradiation treatment for intermediate and advanced laryngeal cancer, there is still work to be done to demonstrate that the long-term functional outcomes of voice and swallowing are better than those achieved by surgery.

Given this range of significant changes in practice over the last 10 years, there are reasons to believe that the reported increase in survival of 3.3% every 5 years is real. Support for the data of Coleman *et al* comes from recently published audit data showing a statistically significant 12.6% improvement in 2-year survival between cohorts of patients in the south and west of England between 1997 and 2000 (Birchall *et al*, 2004).

It is, of course, difficult from these data to identify any particular change in practise that might be the major cause of such improvement in survival. Comparison of detailed Scottish and south and west of England cohorts (MacKenzie and Birchall, unpublished data), totalling 4000 head and neck cancer patients over the same 1998–2002 period showed wide differences in care processes for laryngeal cancer between the two regions, but this did not appear to influence survival. The cure rates for early-stage larynx cancer are already greater than 90% so it is unlikely that improvements in this subset can be responsible for the improvement seen overall. Therefore, we hypothesise that any increase in survival is more likely to devolve from those with advanced disease.

Improved standards of diagnosis, particularly the use of Hopkins' rod telescopes as part of panendoscopy, cross-sectional imaging with CT or MRI, and fine needle aspiration, are likely to have increased the accuracy (and indeed recording) of staging of larynx cancer. These methods allow the identification of more advanced stage disease at the primary site or in cervical lymph node metastases, possibly resulting in ‘stage shift’, with an apparent improvement when none has really taken place (Feinstein *et al*, 1985) Finally, the increased use of specialist multidisciplinary team working, and concentration of care in fewer centres, may have led to better treatment decision making and consequently better outcomes (Bradley *et al*, 2005; Rachet *et al*, 2008).

## The increasing socio-economic gap

Rachet *et al* (2008) describe how difference between most and least deprived groups increased more for laryngeal cancer than for any other cancer in men, and more quickly (almost 4% every 5 years), between 1985 and 2001, reaching 17% for the 1996–1999 cohort. In fact, the most affluent groups accounted for nearly all of the overall increase in survival by themselves.

As other smoking-related cancers were analysed using identical techniques in the present series of reports, it is unlikely that this is either a methodological artefact, or due to some systematic effect of changes in smoking patterns. We are therefore left to consider those factors that are uniquely different about laryngeal cancer and how socio-economic status may affect them.

The care pathway for laryngeal cancer is especially complex; therefore better continuity of care, from GP onwards, may play a part. Better educated people may have better contact with GP's in the first place (Reid and Rozier, 2006) and be better able to negotiate their way through a complex and lengthy pathway of diagnosis, treatment and rehabilitation. Head and neck cancer care, for all the circumstantial evidence in favour of centralisation, remains the most disseminated of all cancers in the United Kingdom. As access to information (and use of the internet particularly) became more widespread during the 1990s, it may be that more affluent patients were better able to choose their hospitals and doctors and to travel to bigger centres. The other unique aspect of laryngeal cancer is that it presents, generally, with a change in voice. Some occupations, professional voice users, may be more sensitive to the symptoms of an early cancer and this may lead to earlier presentation. The remarkable socio-economic effect is not just of passing interest, however. By studying how it really does influence survival for this group, we may learn some crucial lessons about how we organise our care for laryngeal cancer patients in the United Kingdom.

## Conclusions

The observed improvements in survival for laryngeal cancer over the studied period probably are real, and multifactorial in origin. Improvements in accuracy of diagnosis and staging have certainly occurred. Major changes in individual treatment approaches, better team working between surgeons and radiotherapists, and the increased use of combined modality therapy in advanced stage disease are all likely to have played a part. However, the concentration of this improvement in the more affluent groups only suggests some more fundamental effects on survival, which may require substantial (and urgent) changes in the way the diagnostic and care pathways for laryngeal cancer are constructed. Further, prospective studies to examine the contributions of each of these factors are required to optimise management strategies and patterns of care for the future.
